# Directed Assembly of Particles for Additive Manufacturing of Particle-Polymer Composites

**DOI:** 10.3390/mi12080935

**Published:** 2021-08-07

**Authors:** Soheila Shabaniverki, Jaime J. Juárez

**Affiliations:** 1 Department of Mechanical Engineering, Iowa State University, Ames, IA 50011, USA; soheilas@iastate.edu; 2 Center for Multiphase Flow Research and Education, Iowa State University, Ames, IA 50011, USA

**Keywords:** directed assembly, additive manufacturing, external fields, 3D printing, particle-polymer composites

## Abstract

Particle-polymer dispersions are ubiquitous in additive manufacturing (AM), where they are used as inks to create composite materials with applications to wearable sensors, energy storage materials, and actuation elements. It has been observed that directional alignment of the particle phase in the polymer dispersion can imbue the resulting composite material with enhanced mechanical, electrical, thermal or optical properties. Thus, external field-driven particle alignment during the AM process is one approach to tailoring the properties of composites for end-use applications. This review article provides an overview of externally directed field mechanisms (e.g., electric, magnetic, and acoustic) that are used for particle alignment. Illustrative examples from the AM literature show how these mechanisms are used to create structured composites with unique properties that can only be achieved through alignment. This article closes with a discussion of how particle distribution (i.e., microstructure) affects mechanical properties. A fundamental description of particle phase transport in polymers could lead to the development of AM process control for particle-polymer composite fabrication. This would ultimately create opportunities to explore the fundamental impact that alignment has on particle-polymer composite properties, which opens up the possibility of tailoring these materials for specific applications.

## 1. Introduction

Additive manufacturing (AM) is a method by which three-dimensional computer-aided designs (CAD) are fabricated without the need for process planning [1]. This process involves the layer-by-layer deposition of filament, powder, or liquid drops to translate the CAD drawing into a fully realized object. The AM industry generated a revenue of approximately 5.2 billion USD in 2015 [2]. This revenue includes a 769 million USD AM materials market that produces, in descending order of market share, photopolymers, laser-sintered powders, filaments, and metals. These materials are used to support a variety of AM technologies [3] that include 3D printing, stereolithography, laminated object manufacturing, and fused deposition modeling.

Among the materials available for use in AM applications, polymers represent 51% of the products produced by AM systems [2,4]. This fraction does not include the 29% of products that blend polymers and metals together to form composite AM materials. The ubiquity of polymers in the AM industry likely arises from their low cost and high production volume. The U.S. National Research Council reports that polymers, in the form of plastics, fibers, and rubber, are produced in the range of 71 billion pounds per year with a financial return approaching 0.50 USD per pound [5]. As a fundamental building block for AM applications, polymers have properties that can be tuned to achieve specific rheological behavior [6,7,8] or functionalities related to ionic conductivity [9,10,11,12], thermal conductivity [13,14,15], and magnetic [16,17,18] or dielectric [19,20,21] permittivity.

While some of these polymer functionalities can be achieved through chemical modification, an alternative method is to disperse filler micro- or nanoparticles in the polymer solution prior to processing or deposition by AM [22,23,24,25,26]. Inks based on particle-polymer dispersions can be used in the AM process to create compositionally graded composites [27,28] that serve to enhance the multi-material capabilities of finished products [29]. Tuning the AM process for particle-polymer dispersions influences composite properties such as flexibility, mechanical strength, toughness, and optical transparency [30,31,32]. These material properties are directly tied to the filler particle organization within the composite [33]. 

Directing the assembly of filler particles in a composite toward specifically organized arrangements or orientations enables the fabrication of materials which exhibit hierarchical functionality from the nanoscale to the macroscale [34]. Polymer composites that contain aligned filler particles exhibit increased elastic moduli as compared with composites where the filler particles are randomly arranged [35,36,37,38]. Particle-polymer composites with internal alignment also exhibit anisotropic responses to mechanical, thermal, electric, or magnetic inputs [39,40,41,42]. The anisotropy exhibited by the aligned particle-polymer composites can be used for a variety of emerging soft material applications, including wearable sensing devices [43,44,45], energy storage [46,47,48], artificial muscles [49,50,51], and soft robotics [52,53,54]. The alignment of filler particles used in these materials is often achieved by the application of external fields such as electric [55,56], magnetic [57,58], and acoustic fields [59,60,61].

**Figure 1:**
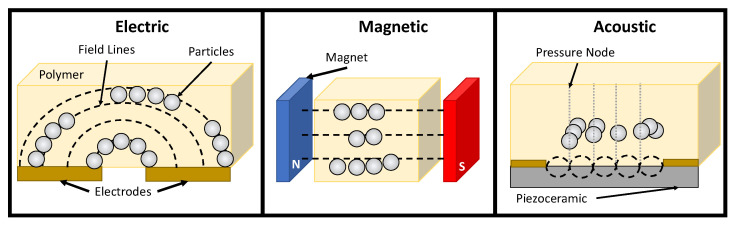
The diagram shows the three major types of external field mechanisms used in AM processing of particle-polymer composites.

It is the purpose of this review article to provide an overview of some of the types of external fields used to direct the assembly or alignment of filler particle-polymer dispersions. Figure 1 summarizes the transport mechanisms discussed in this review. Using external fields to tune the internal arrangement of filler particles is a pathway towards fabricating functionally graded particle-polymer composites. Achieving specific arrangements of filler particle distributions requires an understanding of how different directed assembly mechanisms operate. The forces associated with each of these mechanisms can favor different structures and a fundamental understanding of these forces serves as the basis for a paradigm in multi-material AM processing. The review concludes with a discussion of how external fields can contribute to specific filler particle arrangements that enhance intrinsic material properties (e.g., elastic modulus) or can be used as an actuator to improve AM process control.

## 2. Electric Fields

In uniform electric fields, or at low frequencies, particle transport is dominated by electrophoresis, which represents the migration of a charged particle towards an electrode of opposite polarity. The characteristic electrophoretic velocity as a function of frequency is [62,63]:

(1) $$v_{ep}=\left(b\varepsilon_{p}\varepsilon_{o}\zeta E/\eta m_{p}\right){\left[{\left(b/m_{p}\right)}^{2}+\omega^{2}\right]}^{-0.5},$$

where *b* is the Stokes drag factor, *ε_p_* is the dielectric constant of the particle, *ε_o_* is the electric permittivity of free space, ζ is the particle zeta potential, *E* is the applied electric field, *η* is the medium viscosity, *m_p_* is the particle mass, and *ω* is the angular frequency of the field. Electrophoresis is frequently used to deposit colloidal particles onto surfaces to create functional materials by a process known as electrophoretic deposition [64,65]. 

In one demonstration of electrophoretic deposition for fabricating particle-polymer composites, poly [3-(3-*N*,*N*-diethylaminopropoxy)thiophene] (PDAOT) was dissolved in an acetic acid, water, and ethanol mixture [66]. The mixture was blended with a dispersion of single-wall carbon nanotubes (SWCNTs) and placed in an electrochemical cell, where a 3–15 V direct current (DC) potential was applied over a period of time that varied from 1 to 10 min. The process was used to control the particle-polymer deposition rate for the fabrication of metal oxide nanoparticle-polymer films with a thickness that varied from 100 nm to 15 μm [67,68]. Another polymer, poly (3-octylthiophene) (P3OT), has been blended with SWCNTs to fabricate particle-polymer films with enhanced current density [69]. In these experiments, a 40 V DC potential was applied to create a control particle-polymer film. An alternating current (AC) signal was superimposed onto the input DC signal, resulting in a 12% improvement to current density capacity. The current capacity of electrophoretically deposited particle-polymer films was used as the basis for a sensor which measured changes in resistivity due to in-plane shear stress [70].

Electrodes can also be patterned using photolithography to better concentrate deposition material. Electrode patterning allowed for the fabrication of a conductive particle-polymer film that resulted in a 344% improvement of current density as compared with films fabricated using a DC potential [69]. Photolithography has also been used as part of a process to create a substrate where electrophoretic deposition was applied to Ni fibers dispersed in a polyurethane-modified epoxy resin [71]. An unmodified resin was measured, and its Young’s modulus was 285 MPa after curing, while an electrophoretically deposited Ni fiber resin composite’s Young’s modulus was 6.8 GPa. 

When a non-uniform electric field is used, particles migrate towards field minima or maxima depending on particle and medium properties [72,73,74]. This mechanism, known as dielectrophoresis, affects particles with a force that depends on the gradient of the applied electric field [75]:

(2) $$F_{dep}=\frac{3}{2}\varepsilon_{m}\varepsilon_{o}V_{p}f_{cm}\nabla E^{2},$$

where *ε_m_* is the medium dielectric constant, *V_p_* is the particle volume, and *f_cm_* is the Clausius–Mossotti factor. The parameter, *f_cm_*, is a factor that compares the relative contribution of particle and medium properties to the force acting on the particle. This contribution is as follows:

(3) $$f_{cm}=Re\left[\left({\tilde{\varepsilon }}_{p}-{\tilde{\varepsilon }}_{m}\right)/\left({\tilde{\varepsilon }}_{p}+2{\tilde{\varepsilon }}_{m}\right)\right],$$

where the operator, *Re*, is used to find the real component of the Clausius–Mossotti term and $\tilde{\varepsilon }=\varepsilon \varepsilon_{o}-i\sigma /\omega$ is the complex dielectric permittivity of the particle or the medium. In the DC limit, where *ω* = 0, *f_cm_* is dominated by conductivity values, while dielectric constants are important at high frequencies ($\omega \underset{}{\to }\infty$) [76].

Dipolar chains also form in response to electric field-induced polarization of the particles in solution. The dipolar chain energy acting on a single pair of particles in an electric field is [77]:

(4) $$u_{dc,E}=-\frac{p^{2}}{2\pi \varepsilon_{m}\varepsilon_{o}r^{3}}P_{2}\left(cos\theta \right),$$

where $p=3\varepsilon_{m}\varepsilon_{o}V_{p}f_{cm}E$ is the dipole moment of the particle pair, *r* is the center-to-center separation distance between particle pairs, *P*_2_ is a Legendre polynomial of order two, and *θ* is the angular orientation of the particle pair with respect to the direction of the applied electric field. The force representation of Equation (4) is [78]:

(5) $$F_{dc,E}=-\frac{p^{2}}{4\pi \varepsilon_{m}\varepsilon_{o}r^{4}}\left[\left(15\;cos^{2}\theta -3\right)e_{r}-\left(6\cos\theta \right)e_{z}\right],$$

where ***e_r_*** is a unit vector that connects the particle pair and ***e_z_*** is a unit vector that points in the direction of the electric field.

**Figure 2:**
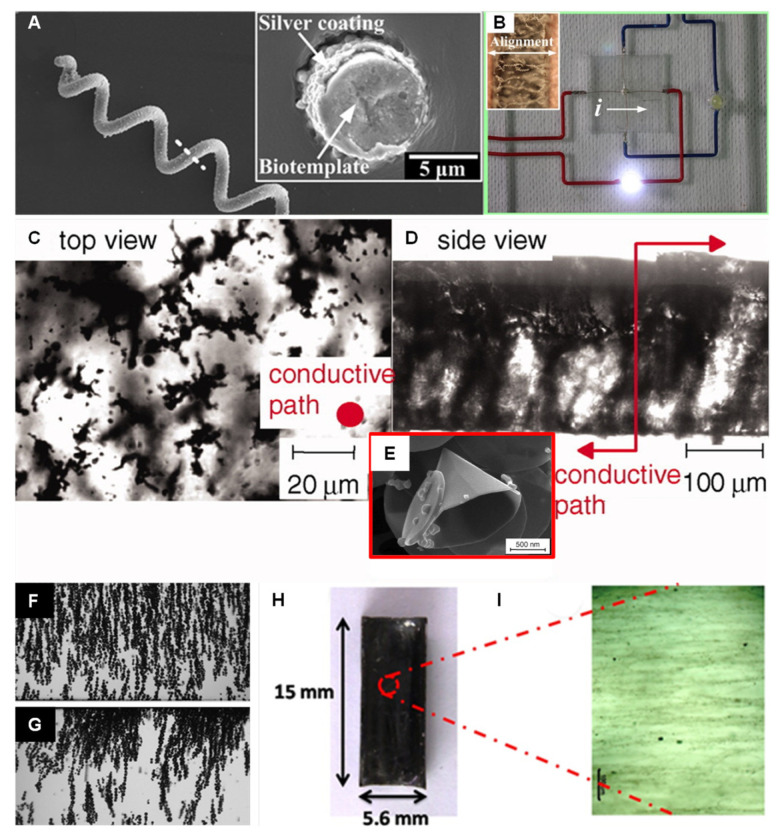
(**A**) SEM image of a metallic Spirulina-templated microcoil. The inset shows a cross-section of the microcoil particle; (**B**) the application of a 5 wt% electric field-aligned microcoil/PDMS composite in a functional LED integrated circuit. Reprinted with permission from [79], copyright 2017 American Chemical Society; optical micrographs showing (**C**) the top view and (**D**) side view of a thermally cured carbon nanocone film (0.2 vol%), which was assembled by DC field alignment; (**E**) inset shows an SEM image of the nanocone used in this process. Reprinted with permission from [80], copyright 2011 John Wiley and Sons; (**F**) hollow glass microspheres dispersed in Norland optical adhesive initially chain before (**G**) migrating towards an electrode. Reprinted with permission from [81], copyright 2017 Elsevier; (**H**) an acrylate polymer composite consisting of (**I**) SWCNT aligned using an AC electric field. Reprinted with permission from [82], copyright 2014 John Wiley and Sons.

In uniform electric fields or electric fields with weak gradients, dipolar chain structures are observed in the resulting composite. These dipolar chains assemble with structures oriented in the primary direction of the applied electric field and, with particle loadings in sufficiently high concentrations, bridge the gap between electrodes, forming a percolated network. *Spirulina*, a microscopic organism with a spring-like structure, is coated with silver and dispersed in polydimethylsiloxane (PDMS) [79]. The *Spirulina* structures are used to create microcoils, as shown in Figure 2A. These microcoil particles assemble into chains between electrodes to form a composite with a conductivity of 10 S/m, which is eight orders of magnitude larger than non-aligned *Spirulina* samples (Figure 2B). Carbon nanocones dispersed in an acrylated urethane form chain structures that improve the electrical conductivity of the polymer from 10^−7^ to 10^−3^ S/m [80]. 

Particle-polymer composites fabricated using electric field assembly can be used to fabricate tactile force sensors. For example, carbon nanocones dispersed in a UV curable adhesive, 3094 Dymax, were assembled into a wire that connects two electrodes spaced 100 μm apart, as shown in Figure 2D [83]. The carbon nanocone wire formed as part of the composite was found to be sensitive to substrate deflections up to 50 μm. CNTs dispersed in PDMS assembled between interdigitated electrodes formed microwires that registered a 0.145% linear change in wire resistivity per unit change in mN of applied force [84]. 

When dielectrophoresis is the dominant transport mechanism, chains form locally in the electric field before migrating towards electric field minima or maxima [74,85]. This can create opportunities to fabricate local concentration gradients in composite materials. The dynamics of particle chain formation in a polymer media under dielectrophoresis mechanism can be predicted using a particle chaining model based on dipole–dipole interactions, which takes into account variable medium viscosity and electric field amplitude [86]. Hollow glass spheres dispersed in Norland optical adhesive initially form chains (Figure 2F) before migrating towards an electrode (Figure 2G) [81]. Barium titanate nanoparticles with a cubic morphology migrate towards electrodes, forming a particle rich layer, when dispersed in PDMS [87]. Anisotropic particles, such as CNTs or fibers, experience an electric field-induced torque before migration towards an electrode, enabling the formation of an aligned nanocomposite (Figure 2H,I) [82,88,89]. The local concentration gradients formed in this way have been used to fabricate composite materials tailored to serve as alternatives to metallic strain gauges [90] or create thermally conductive pathways for enhanced heat transfer [91,92]. 

## 3. Magnetic Fields

Similar as with electric fields, particles respond to magnetic fields by migrating towards magnetic field minima or maxima depending on the intrinsic properties of the particle and the medium in which they are dispersed [93]. The magnetophoretic force responsible for this migration is given as follows:

(6) $$F_{mag}=\frac{\left(\chi_{p}-\chi_{m}\right)V_{p}}{\mu_{o}\left(1+\chi_{m}\right)}\left(B\cdot \nabla B\right),$$

where *χ_p_* and *χ_m_* are the magnetic susceptibilities of the particle and medium, respectively, and *μ_o_* is the magnetic permeability of free space. The magnetic induction, *B*, is related to the applied magnetic field, *H*, by the expression [94] $B=\mu_{o}\left(1+\chi_{m}\right)H$. In addition to magnetophoresis, multiple particles also form dipolar chains in the direction of the magnetic field. The interaction energy between a single pair of particles in a magnetic field is [95]:

(7) $$u_{dc,M}=-\frac{1.202m^{2}}{2\pi \mu_{o}r^{3}}P_{2}\left(cos\theta \right),$$

where $m=\left(\chi_{p}-\chi_{m}\right)V_{p}B/\mu_{o}$ is the magnetic moment of the particle and *θ* is the angle the pair forms with respect to the direction of the magnetic field. The force representation of Equation (7) is [96]:

(8) $$F_{dc,M}=\frac{3\mu_{o}m^{2}}{4\pi r^{4}}\left[\left(1-5\;cos^{2}\theta \right)e_{r}+\left(2cos\theta \right)e_{z}\right],$$

where ***e_r_*** is a unit vector that connects the particle pair and ***e_z_*** is a unit vector that points in the direction of the magnetic field.

The use of magnets as a mechanism for composite fabrication has a long history due to the ease of the process and simplicity of blending para- or ferromagnetic particles with a polymer medium [97,98,99,100,101]. When magnetically sensitive particles are crosslinked in a polymer, the composite forms a magnetoelastomer that deforms in the presence of a magnetic field [102,103]. An alternative method for creating magnetoelastomers was suggested by the authors of this review [104]. In this alternative approach, a sacrificial scaffold was created in PDMS. The scaffold was dissolved, and a magnetorheological fluid was introduced to the evacuated channel, rendering the structure sensitive to magnetic field-induced deflections.

Magnetoelastic soft actuators (sometimes referred to as ferrogels) are designed by coupling magnetic properties of filler particles and elasticity of the polymer matrix. Understanding the deformation and mechanical properties of such actuators is crucial for optimizing their performance [105]. The deformation of these composites in the presence of a magnetic field is analogous to muscle constriction [106], which makes these types of composites ideal for actuation applications such as haptic control surfaces for steering [107]. 

**Figure 3:**
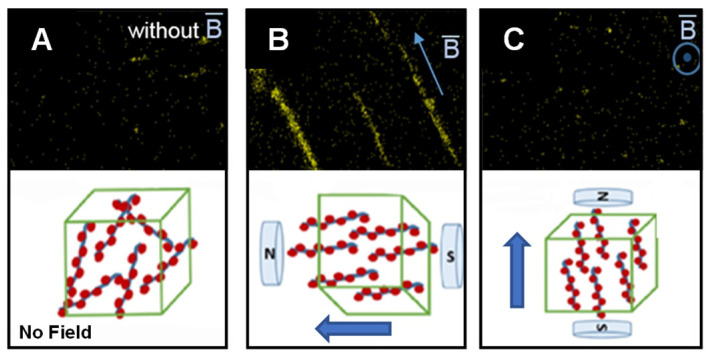
Energy dispersive x-ray images show magnetic cellulose nanocrystals (MGCNC) without (**A**) an applied magnetic field, (**B**) a horizontally aligned magnetic field, and (**C**) a vertically aligned magnetic field. Insets diagrammatically show the field alignment for each case. Reprinted with permission from [113]. Copyright 2011 American Chemical Society.

**Figure 4:**
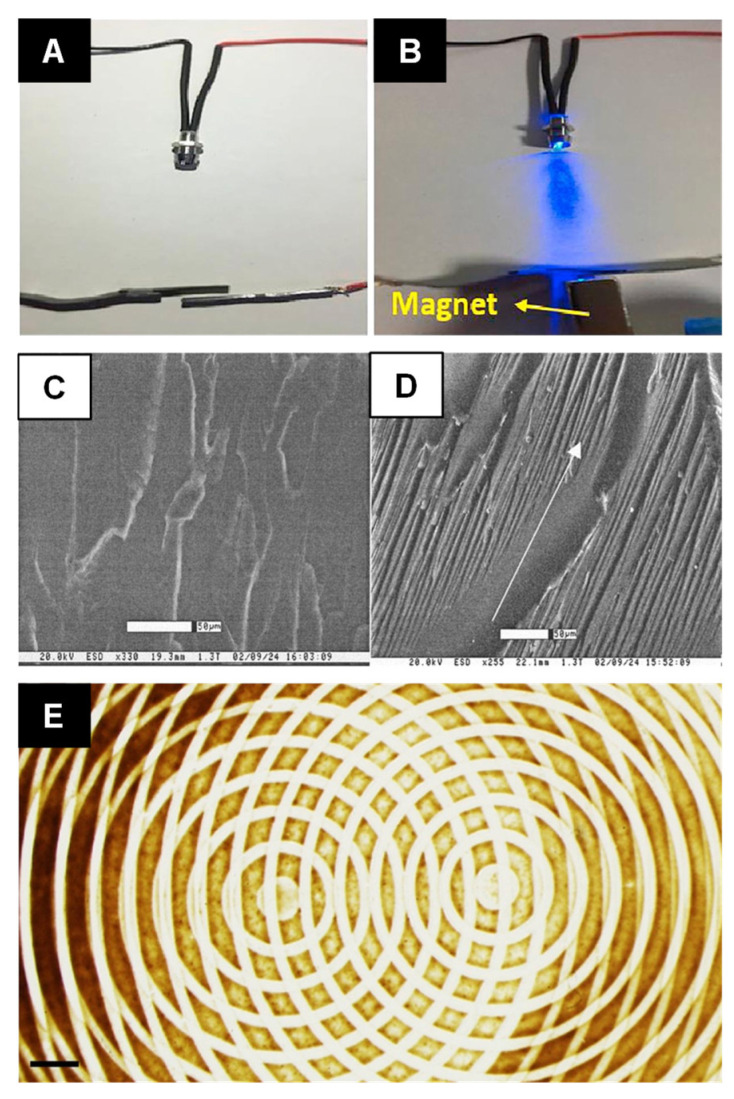
A nanocomposite magnetic switch: (**A**) a gap between an aligned CNT coated composite and a nonmagnetic Pb is (**B**) closed when a magnet is placed underneath the Pb sheet, which lights an LED. Reprinted with permission from [112]. Copyright 2019 Elsevier. Environmental scanning electron microscopy images show the morphology of an epoxy composite sample processed at (**C**) no applied magnetic field and (**D**) at 15 T. The arrow represents the direction of the corresponding magnetic field. Reprinted with permission from [114]. Copyright 2004 John Wiley and Sons. (**E**) A complex pattern formed in a composite by the superposition of two separate magnetic fields. Reprinted with permission from [115]. Copyright 2017 Springer Nature.

Magnetite (Fe_3_O_4_) is one of the most frequently used filler material due to its high magnetic susceptibility [108], which makes it easy to pattern structures in polymers using externally directed magnetic fields [109]. Fe_3_O_4_ can be adsorbed on cellulose nanocrystals to fabricate magnetic cellulose nanocrystals (MGCNCs). The MGCNCs, shown in Figure 3, then are aligned in different configurations (parallel or perpendicular directions) in the polylactic acid matrix through a tunable magnetic field which result in fabrication of particle-polymer nanocomposite with anisotropic electrical and magnetic properties. The percent elongation improvement of the resulting nanocomposite is in the range of 60% to 240% [110]. This use of magnetic fields to locally pattern magnetite-PDMS composites led to a reduction in local elastic modulus by as much as 50% depending on filler particle concentration. While local elastic properties of the composite may see a reduction due to magnetic field-induced concentration, the bulk storage moduli of a magnetite-based composite are observed to increase by a factor of two depending on the field magnitude used to align particles [111]. Magnetite can also be functionally bound to materials that are not ferromagnetic, such as cellulose [112] or glass [113], to create materials with anisotropic mechanical properties or functional structures, like the circuit shown in Figure 4A,B. 

Aside from magnetite, carbon nanotubes (CNTs) are also extensively used with magnetic fields to create aligned composites [116,117,118]. When a magnetic field is applied to anisotropic particle like CNT, the particle rotates with a torque of [119],

(9) $$\tau_{mag}=m\mu_{o}H\;sin\left(\varphi_{f}-\varphi_{p}\right),$$

where *φ_f_* is the angular orientation of the magnetic field and *φ_p_* is the angular orientation of the particle. Multi-walled and single-walled CNTs blended into two separate types of epoxy exhibit different mechanical properties as a function of magnetic field strength [120]. Multi-walled CNTs blended with a bisphenol-A type epoxy (Figure 4C,D) only exhibits a small (~25%) increase in elastic modulus as a function of magnetic field strength, up to 25 T. However, single-walled CNTs increase the elastic modulus by a factor of nearly two when the same alignment process is used. A separate epoxy based on bisphenol-A and bisphenol-F actually exhibit a decline in elastic modulus for both dispersions based on both types of CNTs. This is probably due to the interaction between the magnetic field and the dipole of the polymer [114].

Since the magnetophoretic effect scales volumetrically, strong magnetic fields can induce dipole alignment in both the filler particle and the polymer. Fe_3_O_4_ magnetic nanoparticles were assembled within PMMA film by magnetophoretic forces using two permanent magnets, one was stationary and the other was manipulated in order to create complex interference-like patterns (Figure 4F) [115]. Low-strength magnetic fields are sometimes used to minimize the complex effects that polymer alignment has on the mechanical properties of the composite [121]. The susceptibility of CNTs to low-strength magnetic fields is typically enhanced by blending with iron oxide particles. This blending process allows for filler particle alignment at magnetic field strengths as low as 0.5 T in a rubber medium, which improves the compressive strength by as much as 285% as compared with rubber alone [122]. Another process uses acids to oxidize CNTs and create functional groups, after which the CNTs are placed in a mixture containing ferrous sulfate and iron (III) chloride and stirred together in ammonia to initiate a reaction that attaches magnetite to the functional groups [123]. The magnetite/CNT particles are dispersed in a bisphenol-A (DGEBA)/polyoxypropylenediamine epoxy and cured during exposure to a 0.7 T magnetic field. The composites made from these particles are observed to have a lower crack density than epoxy laminate in the absence of particles, which is ideal for composites that undergo thermal cycling. 

Another process uses surfactants to tether single-walled CNTs to 50 nm γ-iron (III) oxide particles [124]. This process enables a 1–2 wt% nanoparticle-CNT dispersion to assemble into aligned microstructures in an epoxy composed of a bisphenol A polymer. The composites formed by this process exhibit a 9.8% increase in ultimate tensile strength. The enhanced mechanical properties of the composite are brought on by field aligned CNT/iron particles [125]. Anisotropic mechanical properties enable particle-polymer composites based on magnetic alignment to exhibit bending modes that enable the fabrication of soft actuation elements [53].

Aside from mechanical properties, magnetic alignment of particles can also enhance electrical properties of the composite. Magnetite particles aligned in a 25 mT magnetic field have been observed to increase the electric conductivity from 7.14 × 10^−9^ S/m for a randomly dispersed sample to 0.0014 S/m for aligned samples [126]. The enhancement in electric conductivity also extends to magnetite-functionalized CNT particles, where aligned dispersions prepared in a bisphenol A/methylenedianiline epoxy exhibit conductivities of 0.5 S/m [127]. The increase in conductivity is greatest in the direction of particle alignment, while conductivity is reduced in a direction perpendicular to alignment. Aligned particle-polymer composites undergo linear changes in resistance as a function of strain, with applications to mechanical sensing [128].

The process of aligning particles can also lead to enhancements in thermal conductivity. Values of 1.43 W/m-K for thermal conductivity have been observed in the direction of alignment for 40 vol% of magnetite dispersed in epoxy [129]. Similar values of thermal conductivity have been observed for magnetite-coated diamond particles, although only a volume fraction of 15% was necessary in this case [130]. Thermal conductivities of ~5 W/m-K have been observed for aligned nickel particles in a polymer matrix, which represented an increase of as much as 175% over the polymer alone [131]. Composites formed from CNTs dispersed in a polyvinyldene fluoride matrix have exhibited thermal resistances as low as 11 × 10^−9^ m^2^K/W [132]. Modification of thermal conductivity by filler particle assembly can be used to improve thermal interfacing between surfaces [133] or retard thermal transport [134].

## 4. Acoustic Fields

An acoustic source, such as a piezeoelectric transducer, generates an acoustic radiation force that can be used to transport particles. The acoustic radiation force responsible for transporting particles is dependent on the gradient of the pressure field generated by the acoustic source [135,136,137]:

(10) $$F_{ac}=\frac{\pi \beta_{m}V_{p}\Phi }{2\lambda }\nabla P^{2},$$

(11) $$\Phi =\frac{5\rho_{p}-2\rho_{m}}{2\rho_{p}+\rho_{m}}-\frac{\beta_{p}}{\beta_{m}},$$

where *β* = 1/*ρc*^2^ is the compressibility, *ρ* is density, *c* is the speed of sound, *Φ* is the acoustic contrast factor, *λ* is the wavelength of the acoustic field, and *P* is the pressure distribution. The subscripts *p* and *m* refer to particle and medium properties, respectively. The pressure distribution for a one-dimensional standing wave is [138] $P=P_{o}cos\left(2\pi fx/c_{m}\right)$, where *P_o_* is the pressure amplitude and *f* is the applied frequency.

Acoustic nodes are created when the acoustic wave is reflected. The resulting superposition of incident and reflected waves interfere with each other [139]. This phenomena, known as standing acoustic waves (SAW), has long been recognized as a method for separating small particles or drops dispersed in a fluid [135,140,141,142]. The relative difference in particle density and compressibility dictates whether particles migrate towards or away from the acoustic pressure node where the wave interference leads to a local pressure of zero. Particles that are more dense and less compressible than the medium, such as polystyrene [143] or cells [144], migrate to acoustic pressure nodes. Particles such as microbubbles [145] or PDMS [146] are less dense or more compressible than the medium in which they are dispersed, leading to migration towards the acoustic pressure antinode, where the pressure gradient is highest. 

**Figure 5:**
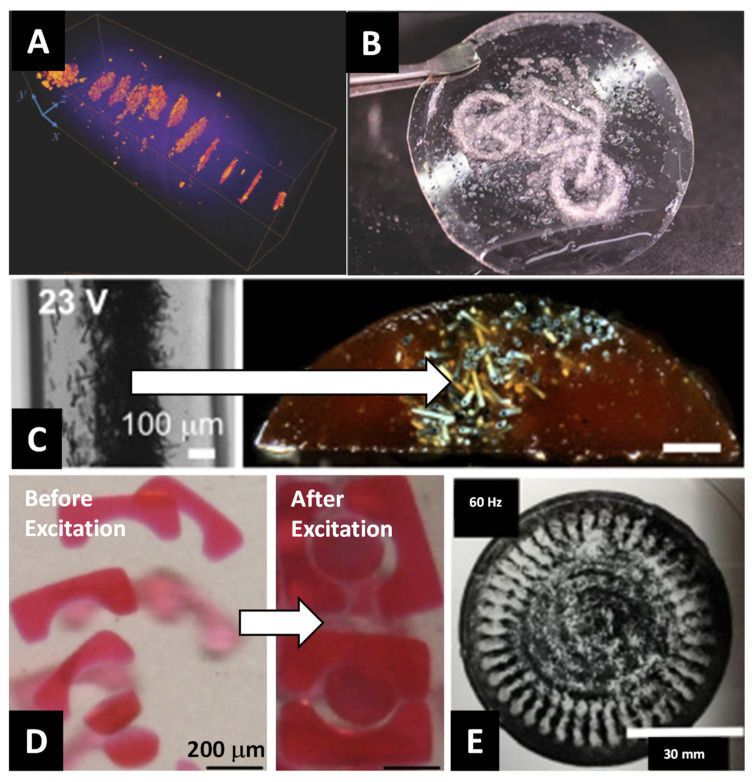
(**A**) An X-ray microtomographic rendering of ~5 nm diamond nanoparticles assembled in an epoxy composite using a 1 MHz ultrasound standing wave. Reprinted with permission from [150], copyright 2011 AIP Publishing; (**B**) holographic tweezers used acoustic fields to assemble silicone particles to form complex, nonsymmetric structures. Reprinted with permission from [153], copyright 2011 John Wiley and Sons; (**C**, **left**) acoustically assembled fibers assembled at an acoustic node in an epoxy; (**C**, **right**) the composite is deposited on a substrate at a rate of 3.7 mm/s to form a printed line. Reprinted with permission from [154], copyright 2016 Elsevier; (**D**) lock (1 mm square) and key (200 μm circle) microgel assemble before excitation (left) and after acoustic excitation (right). Reprinted with permission from [155], copyright 2011 Elsevier; (**E**) iron oxide nanoparticles (50–100 nm) assemble in a hydrogel through vibrational excitation of the medium. Reprinted with permission from [60], copyright 2018 Elsevier.

To demonstrate the utility of SAWs as a method for particle-polymer composite fabrication, early studies used polysiloxane as the polymer medium due to its ease of use and low cost [147]. The composite was prepared by blending the polysiloxane with a curing agent at a ratio of 20:1. Ten-micron acrylic spheres were dispersed in the polysiloxane fluid to act as filler particles. The acoustic field frequency was adjusted to create multiple acoustic nodes for filler particle assembly and allowed to set for 8 h. Adjusting the orientation and number of transducers can lead to different internal microstructures, such as lines or lattices [148]. Diffraction experiments showed that acoustic fields were excellent for localizing particles around the acoustic node [149]. Diamond nanoparticles with diameters of 5 nm were successfully arranged in ethanol-diluted epoxy matrix using acoustic standing wave to produce polymer nanocomposite. The radiation force of standing waves in a rectangular chamber was used to pattern clusters of nanoparticles. During the epoxy curing cycle of 5 min, the standing wave was activated and diamond nanoparticles migrated toward the nodes due to acoustic radiation force and formed quasi-parallel planes of particle clusters (Figure 5A) [150]. Complex particle-polymer composite structures can be formed using SAWs if the acoustic signal is first passed through a pattern that acts like a “hologram” for the desired structure (e.g., Figure 5B) [151,152,153].

Localizing particles at the node has be done by adjusting the input voltage into the piezoelectric transducer, which linearly scales with applied pressure amplitude [156]. Tuning the input voltage controls the degree of particle ensemble compression, which affects the equilibrium particle microstructure during transport within the acoustic field [61,157,158]. This creates opportunities to use SAWs as a method to dynamically control the internal arrangement of filler particles at the acoustic node during 3D printing. For example, a base ink used for 3D printing consisting of a thermally curable epoxy resin and polydisperse glass spheres has been extruded through a printhead with a piezoelectric transducer and demonstrated the fabrication of a log pile structure formed with internally structured particle-polymer composite lines (e.g., Figure 5C) [159]. Multiple lines have been formed during the extrusion process when the ink was blended with negative and positive acoustic contrast particles [154]. SAWs has also been used during the sterolithography process where piezoelectric transducers could be attached to a vat holding a photocurable polymer to hold particles in place during the curing phase [160]. Multiple piezoelectric transducers were used during the stereolithography process to assemble CNTs that enhanced the electric conductivity of the resulting particle-polymer composite [161].

SAWs can also be used to orient and assemble anisotropic particles. The forces that arise from these mechanisms drive anisotropic particles such as rods, bricks, or bowtie shapes to orient themselves in a direction that is parallel to the acoustic node [162]. These forces have been used to assemble glass fibers dispersed in a dimethacrylate monomer fluid [59,163]. SAWs were used to generate periodic arrays of pressure nodes and antinodes and trap stimulus-responsive coacervate microdroplets to fabricate parallel lines and grid-like micropatterned polymer/dipeptide droplets [164]. Acoustic fields have been used to assemble fibers on a timescale of ~667 ms and photocured with an LED light for a period of 40 s to create a composite that improved the tensile strength by a factor of 43% as compared with a randomly distributed sample. The light source used to cure the sample could be mounted to a 3D printer gantry to locally cure the acoustically assembled fiber-polymer composite [165]. Acoustic fields have also been applied to assemble CNTs in a thermosetting polymer resin, which improves the ultimate tensile strength by 6% and the elastic modulus by 51% [166].

SAWs have been used to fabricate biological composites due to observations that cells do not appear to exhibit long-term effects because of acoustic field exposure [167,168]. Yeast cells dispersed in acrylamide or alginate gels assembled at acoustic nodes in 10 min and cured in approximately 1 min after the assembly process was complete [169,170]. The cells concentrated at the acoustic node remodeled the surrounding gel matrix where the cells locally caused the gel network to contract [171]. The process of concentrating cells at an acoustic node enabled the study of cell–cell interactions, which was not possible for isotropically distributed cells. A live/dead assay performed on acoustically assembled biocomposites showed that approximately 50% of the cells in the sample remained viable after 40 h [172]. The concentrated cell structures formed at acoustic nodes formed sprouts that were nearly three times longer than those formed in samples not directly exposed to the SAW, indicating the formation of vasculature [173]. Cells seeded in isolated hydrogel beads assembled into complex 3D structures (Figure 5D) that could serve as model tissues for a variety of biotechnology applications [155]. Alternative approaches for creating hydrogel-based biocomposites have relied on modification of the AM platform to assembly cells during the extrusion process [174] or to break the surface tension at the nozzle tip to create tunable hydrogel droplets [175].

An alternative method for transmitting acoustic waves is to have the waves pass through a substrate rather than into the fluid directly. This type of wave is known as a surface standing acoustic wave (SSAW) and the acoustic radiation force experienced by particles remains the same as the one presented above [176]. A chamber placed on top of a vibration generator was used to produce SSAWs that directed the assembly of cellular spheroid colonies consisting of mouse fibroblasts dispersed in crosslinked fibrinogen [177]. The same method was applied to assemble cardiomyocytes into a model cardiac tissue [178]. Cardiomyocytes assembled in this way exhibited 2.5 times greater contractile stress and faster beating after 5 days as compared with randomly distributed samples. SSAWs can also be generated using electrodes patterned on a piezoelectric substrate, which was used to assemble cardiomyocytes in a photocurable gelatin [179]. The cardiomyocytes exhibited beating in 5 to 7 days after assembly. 

The patterns observed in samples assembled using SSAWs resemble Chladni figures [180], which result from time-varying localized flexing of the substrate. This flexing causes the appearance of acoustic nodes, which correspond to plate displacement anti-nodes (i.e., where displacement is maximal or minimal) [181]. A comparison of microparticle and nanoparticle assembly in a PEGDA hydrogel showed that microparticles preferentially migrate to the acoustic nodes, while the nanoparticles migrate away from the acoustic nodes [60]. A scaling analysis of this phenomena showed that nanoparticles were likely entrained in an acoustically generated flow known as acoustic streaming [182]. Assuming spherical geometry, the characteristic acoustic streaming velocity is [183]:

(12) $$v_{str}=\frac{3}{16}\frac{P_{o}^{2}}{\rho_{m}^{2}c_{m}^{3}}sin\left(2kx+N\pi \right),$$

where *m* is the solvent viscosity, *k* = *ω*/*c_m_* is the wave number, *ω* is the acoustic field frequency, and *N* = *ω w*/*π c_m_* is the number of acoustic nodes in a system with a characteristic width of *w*. To the best of our knowledge, the work by Shabaniverki et al. [60], shown in Figure 5E, is the only demonstration of acoustic streaming as a transport mechanism applied to AM. 

## 5. Future Directions and Conclusions

**Figure 6:**
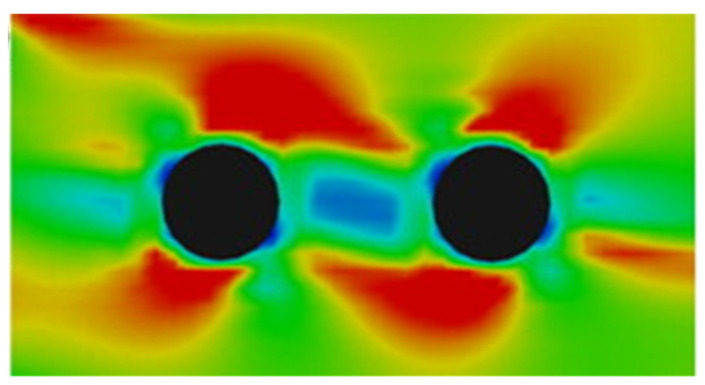
Simulation of a sheared particle pair in a viscoelastic fluid. The stress in the fluid favors chaining the direction of shear. Reprinted with permission from [196], copyright 2014 Elsevier.

Applying external fields to particles dispersed in a polymer solution creates opportunities to target specific mechanical properties by tuning particle microstructure through alignment. Halpin and Tsai were the first to develop models that described the mechanical properties of particles aligned in a composite [184,185]:

(13) $$\frac{E}{E_{o}}=\frac{1+\eta \phi }{1-\eta \phi },$$

(14) $$\eta =\frac{\frac{E_{p}}{E_{o}}-1}{\frac{E_{p}}{E_{o}}+\xi },$$

(15) $$\xi =\frac{L}{a},$$

where *E_o_* is Young’s modulus of the polymer in the absence of particles, *E_p_* is Young’s modulus of the particle phase, *ϕ* is the particle volume fraction, *L* is the length of the particle, and *a* is the particle’s radius. While the Halpin–Tsai model is frequently used to analyze composites with aligned fillers, it suffers from several deficiencies [186,187]: (1) It is a semi-empirical model that does not provide any insight into particle–particle interactions and (2) it assumes that all the constituent particles are perfectly aligned. In order to overcome some of these limitations, it is necessary to incorporate the statistical distribution of particles through a parameter, such as the radial distribution function (RDF). The RDF is a probability density function that describes the distribution of particles in the sample as a function of particle pair separation. Song and Zheng [188] suggested an expression originally developed by Verberg et al. [189]:

(16) $$\frac{E}{E_{o}}=\chi \left[1+\frac{1.44\phi^{2}\chi^{2}}{1-0.1241\phi +10.46\phi^{2}}\right],$$

where *χ* is the RDF at contact between particles. This expression was originally derived from a statistical mechanics analysis of hard spheres dispersed in a Newtonian fluid. However, the polymers used in AM typically exhibit viscoelastic behavior [190]. Experimental observations of particles dispersed in a viscoelastic fluid flowing in a tube show that particles may preferentially migrate towards channel walls [191,192]. Viscoelasticity can also lead to particles chaining when subject to applied shear [193,194,195]. The stress distribution around individual particles causes particles to assemble into chains when sheared in a viscoelastic fluid (Figure 6) [196]. Additional work is needed to clarify the role that viscoelasticity plays in determining particle distribution in shear flow, particularly, how it applies to AM.

While the model described by Equation (16) is incomplete, it does offer some insight that is not available from the Halpin–Tsai model. Equation (16) includes particle interactions (i.e., hard sphere) and incorporates particle distribution in the form of the RDF. Limitations include the assumption that particle distribution is isotropic, which would not be true for field aligned particles and is reportedly valid for bulk volume fractions below 0.55, which precludes its use in modeling the AM of solidified structures, such as colloidal crystals [197]. Measurements performed by Akella et al. on 3D printed particle-polymer fibers showed that the model described by Equation (13) was consistent for samples with random distributions of particles [198]. However, when an acoustic field was used to assemble the particles during printing, the fiber printed by Akella et al. exhibited an increase in χ due to improved particle packing. More experimental work is necessary to help elucidate the impact that RDF has on composite mechanical properties. Another potential avenue of research is the development of analytical expressions that better describe composite mechanical properties and their dependency on particle alignment and RDF.

**Figure 7:**
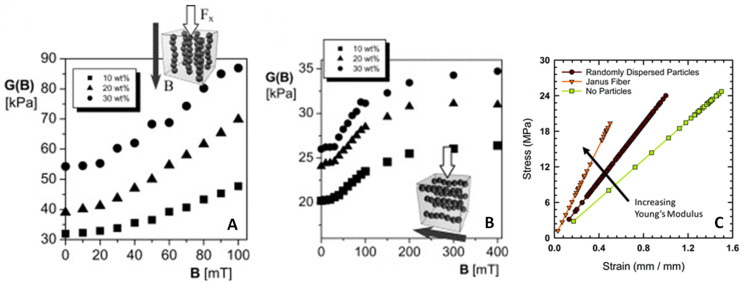
Elastic moduli for a composite of magnetically aligned particles with the load applied: (**A**) In the direction of alignment; (**B**) in the transverse direction of alignment. Reprinted with permission from [35], copyright 2006 Elsevier. (**C**) Stress-strain curves for a 3D printed fiber without particles (squares), with randomly distributed particles (circles), and acoustically assembled particles (triangles). Reprinted with permission from [198], copyright 2020 Royal Society of Chemistry.

The mechanical properties of composites with external field aligned particles have been shown to exhibit an orientational dependence, as shown in Figure 7A,B [35,199]. We can qualitatively observe this dependence by introducing an orientation parameter, *θ*, to the RDF, which is the same as the direction of alignment from Equations (4) and (7) [200]:

(17) $$\chi \approx \chi_{hs}\;exp\left(\Lambda \left[\frac{3\;cos^{2}\theta -1}{2}\right]\right),$$

where *χ_hs_* is the RDF at contact for hard spheres [201], $\chi_{hs}=\left(1-0.5\phi \right){\left(1-\phi \right)}^{-3}$, and *Λ* is a dimensionless field energy density, which are given in Table 1 for the mechanisms described in this review. The effect of the field-driven particle alignment on mechanical strength can also be seen in Akella et al. [198], who found that the Young’s modulus increased from 24.7 MPa for the fiber without particles to 50.5 MPa for a fiber with acoustically assembled particles.

To qualitatively see how RDF impacts mechanical properties, consider the cases of parallel (∥, *θ* = 0°) alignment:

(18) $$\chi_{∥}\approx \chi_{hs}\;exp\left(\Lambda \right),$$

**Figure 8:**
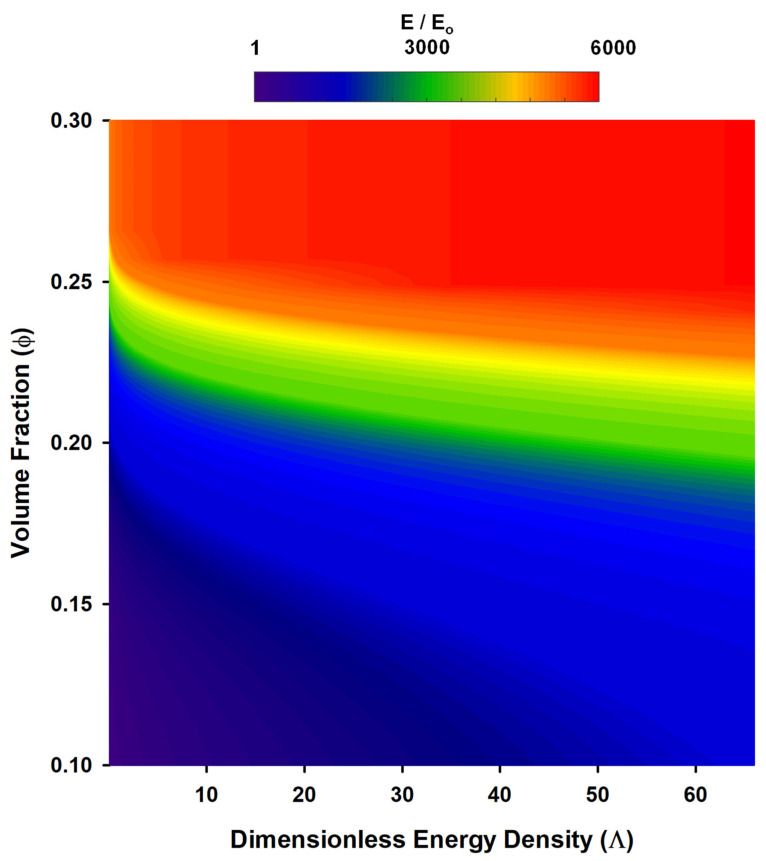
A contour plot of normalized Young’s modulus (Equation (16)) illustrating the impact that filler particle volume fraction and the dimensionless energy density of an applied external field have on mechanical properties. The field influences the RDF of the filler particles (Equation (18)), which also influences Young’s modulus as described by Equation (16).

Thus, we can see that parallel alignment has a strong effect on RDF. According to Baxter-Drayton and Brady [200], the RDF at contact for an aligned system saturates at high values of *Λ*, limiting $\chi_{∥}$ to a maximum value of ~240. Qualitatively, based on Equation (16), this translates to a larger elastic modulus in the direction of alignment. We combined Equations (16) and (18) to produce Figure 8, which shows how RDF impacts Young’s modulus value. A combination of field strength, measured by Λ, and volume fraction, potentially, can increase Young’s modulus (*E_o_*) by as much as 6000 times that of bare polymer. To illustrate the significance of this, consider that a polymeric material, such as PDMS, has a Young’s modulus of ~2 MPa [209]. The inclusion of field aligned particles could increase the elastic modulus of PDMS to a value of ~12 GPa, which is comparable to bone, concrete, or certain types of wood. Overall, the connection between particle distribution vis-à-vis RDF and mechanical properties serves as another possible avenue for future research. While the model presented above suggests a connection to mechanical properties, it would also be useful to explore how RDF impacts other particle-polymer composite properties, such as thermal [210,211,212] or electrical [213,214,215] conductivity. 

Models of particle behavior during the AM process could lead to process control techniques that affect field conditions (e.g., amplitude and frequency) [74,216,217], particle type [218,219,220], and shear stress [221,222,223]. A fundamental understanding obtained through modeling would be a pathway towards achieving specific microstructures with targeted properties. Such process control strategies could be developed using real-time field actuation to direct particle assembly [224,225,226], gain-scheduling policies that maximize particle packing [227,228,229], or the identification of an open-loop strategy that would achieve a desired order parameter, such as the degree of hexagonal close-packing [230,231].

Applying external fields to AM of particle-polymer composites provides a unique approach to functionally structure these composites by internally aligning the particles. This would create opportunities to fabricate these composites for targeted mechanical, thermal, electrical, or optical applications. However, it is necessary to improve our fundamental understanding of the AM process in the presence of an external field and how composite processing impacts its material properties. Process control for directed assembly-based AM is an area of research that requires further exploration. Challenges associated with this line of research include identifying the appropriate transport mechanism for a given polymer/particle system to use as an actuator, developing sensing techniques that could be used to guide the assembly process in real time, and connecting the composite properties to match desired material properties.

## Figures and Tables

**Table 1:** Dimensionless energy density for the mechanisms examined in this review.

Field Type	*Λ* ^1^	References
Electric	$\pi \varepsilon_{m}a^{3}{\left(f_{cm}E_{o}\right)}^{2}/k_{B}T$	[85,202,203]
Magnetic	$\pi \mu_{o}a^{3}{\left[\left(\chi_{p}-\chi_{m}\right)H\right]}^{2}/9k_{B}T$	[95,204,205]
Acoustic	$V_{p}\Phi^{2}E_{ac}/2k_{B}T$	[206,207,208] ^2^

^1^ The dimensionless energy density is scaled by, *k_B_*, Boltzmann’s constant and the ambient temperature, *T*. ^2^ $E_{ac}=P_{o}^{2}/4\rho_{m}c_{m}^{2}$ is the acoustic energy density. The one-half factor comes from the fact that *E_ac_* represents the full oscillation range for the acoustic field. Thus, *E_ac_*/2 represents the acoustic field energy density magnitude.

## Data Availability

All data provided in the present manuscript are available to whom it may concern.
